# *GRIN2A* Variants Associated With Idiopathic Generalized Epilepsies

**DOI:** 10.3389/fnmol.2021.720984

**Published:** 2021-10-14

**Authors:** Xiao-Rong Liu, Xing-Xing Xu, Si-Mei Lin, Cui-Ying Fan, Ting-Ting Ye, Bin Tang, Yi-Wu Shi, Tao Su, Bing-Mei Li, Yong-Hong Yi, Jian-Hong Luo, Wei-Ping Liao

**Affiliations:** ^1^Key Laboratory of Neurogenetics and Channelopathies of Guangdong Province and the Ministry of Education of China, Institute of Neuroscience and Department of Neurology of the Second Affiliated Hospital of Guangzhou Medical University, Guangzhou, China; ^2^Department of Physiology, Wenzhou Medical University, Wenzhou, China; ^3^NHC and CAMS Key Laboratory of Medical Neurobiology, MOE Frontier Science Center for Brain Research and Brain-Machine Integration, School of Brain Science and Brain Medicine, Zhejiang University School of Medicine, Hangzhou, China

**Keywords:** *GRIN2A* gene, *N*-methyl-D-aspartate receptors, gain of function, sub-regional effect, idiopathic generalized epilepsy

## Abstract

**Objective:** The objective of this study is to explore the role of *GRIN2A* gene in idiopathic generalized epilepsies and the potential underlying mechanism for phenotypic variation.

**Methods:** Whole-exome sequencing was performed in a cohort of 88 patients with idiopathic generalized epilepsies. Electro-physiological alterations of the recombinant *N*-methyl-D-aspartate receptors (NMDARs) containing GluN2A mutants were examined using two-electrode voltage-clamp recordings. The alterations of protein expression were detected by immunofluorescence staining and biotinylation. Previous studies reported that epilepsy related *GRIN2A* missense mutations were reviewed. The correlation among phenotypes, functional alterations, and molecular locations was analyzed.

**Results:** Three novel heterozygous missense *GRIN2A* mutations (c.1770A > C/p.K590N, c.2636A > G/p.K879R, and c.3199C > T/p.R1067W) were identified in three unrelated cases. Electrophysiological analysis demonstrated R1067W significantly increased the current density of GluN1/GluN2A NMDARs. Immunofluorescence staining indicated GluN2A mutants had abundant distribution in the membrane and cytoplasm. Western blotting showed the ratios of surface and total expression of the three GluN2A-mutants were significantly increased comparing to the wild type. Further analysis on the reported missense mutations demonstrated that mutations with severe gain-of-function were associated with epileptic encephalopathy, while mutations with mild gain of function were associated with mild phenotypes, suggesting a quantitative correlation between gain-of-function and phenotypic severity. The mutations located around transmembrane domains were more frequently associated with severe phenotypes and absence seizure-related mutations were mostly located in carboxyl-terminal domain, suggesting molecular sub-regional effects.

**Significance:** This study revealed *GRIN2A* gene was potentially a candidate pathogenic gene of idiopathic generalized epilepsies. The functional quantitative correlation and the molecular sub-regional implication of mutations helped in explaining the relatively mild clinical phenotypes and incomplete penetrance associated with *GRIN2A* variants.

## Introduction

Idiopathic generalized epilepsies (IGEs) (G40.3 in ICD-10 2016, WHO), also known as genetic generalized epilepsies (GGE, OMIM# 600669), are a group of self-limited epileptic syndromes characterized by recurring generalized seizures without any underlying anatomic or neurological abnormality (Berrin et al., 2015; Scheffer et al., 2017; Collaborative, 2019). Idiopathic generalized epilepsies include juvenile myoclonic epilepsy (JME), juvenile absence epilepsy (JAE), childhood absence epilepsy (CAE), and epilepsy with generalized tonic-clonic seizures alone (EGTCS) (Engel and International League Against Epilepsy [ILAE], 2001). Generally, IGEs were regarded as a group of genetically determined disorders (Mullen and Berkovic, 2018). Monogenic abnormalities only account for 2–8% of IGEs (Weber and Lerche, 2008; Prasad et al., 2013). Exome-based genetic screening studies have demonstrated that over twenty genes were associated with IGEs, such as *CACNA1H*, *CACNB4*, *CASR*, *CHD4*, *CLCN2*, *EFHC1*, *GABRD*, *GABRA1*, *GABRG2*, *GABRB3*, *HCN2*, *KCC2*, *KCNMA1*, *RORB*, *SCN1A*, *SLC12A5*, *SLC2A1*, *RYR*2, and *THBS1* (DiFrancesco et al., 2011; Striano et al., 2012; Kahle et al., 2014; Rudolf et al., 2016; Santolini et al., 2017; Wang et al., 2017; Abou El Ella et al., 2018; Li et al., 2018; Yap and Smyth, 2019; Chan et al., 2020; Liu et al., 2021). Recent studies also identified several copy number variants associated with IGEs, such as duplication at 8q21.13-q22.2 and microdeletions at 1q21.1, 15q11.2, 15q13.3, and 16p13.11 (de Kovel et al., 2010; Kirov et al., 2013; Møller et al., 2013; Jähn et al., 2014; Rezazadeh et al., 2017). Clinically, genetic etiologies in majority of the cases with IGEs remain unknown. On the other hand, although IGEs were generally considered as genetic epileptic syndromes, big pedigrees of IGEs were rare. Variants with incomplete penetrance in IGEs-associated genes are common.

*GRIN2A* gene (OMIM^∗^ 138253), encoding GluN2A subunit of *N*-methyl-D-aspartate receptors (NMDARs), is comprehensively expressed in human cerebral cortex since embryonic period^1^ and plays a critical role in excitatory synaptic transmission, plasticity and excitotoxicity in the mammalian central nervous system (Bar-Shira et al., 2015; Bagasrawala et al., 2017). Previously, *GRIN2A* mutations were found to be mainly associated with idiopathic focal epilepsy with incomplete penetrance (Carvill et al., 2013; Lemke et al., 2013; Lesca et al., 2013) and occasionally with epileptic encephalopathy (EE) (Venkateswaran et al., 2014; Yuan et al., 2014). So far, no *GRIN2A* mutation has been identified in patients with IGEs.

In the present study, trio-based whole-exome sequencing was performed in a cohort of patients with IGEs. Three novel missense mutations in *GRIN2A* gene were identified. Further studies showed that the missense mutations led to gain of function of NMDARs and/or increased membrane protein expression. To understand the underlying molecular mechanism for phenotypic variation, the correlations between the functional alterations and phenotypic severity, and the sub-regional effect of missense mutations were analyzed.

## Materials and Methods

### Subjects

A total of 88 patients with IGEs, including 47 patients with JME, 15 with JAE, 12 with CAE, and 14 with EGTCS, were recruited in Epilepsy Center of the Second Affiliated Hospital of Guangzhou Medical University from February 2013 to December 2018. Patients with IGEs were diagnosed according to the classification of epilepsy and epileptic syndromes by International League Against Epilepsy (Commission on Classification and Terminology of the International League Against Epilepsy, 1989; Engel and International League Against Epilepsy [ILAE], 2001; Helbig, 2015; Scheffer et al., 2017). The collected clinical data included semiology and evolution of the disorders, family history, and the data of treatment. The patients with abnormalities of general and/or neurological examinations were excluded. Video-electroencephalogram (EEG) monitoring recordings that included hyperventilation, intermittent photic stimulation, and sleep recordings were obtained to confirm the diagnosis of IGEs. The patients were included if they had at least one subtype of generalized seizures (including primarily generalized tonic-clonic seizure, myoclonic, and absence seizure) but no partial seizure. Their electroencephalogram (EEG) was characterized by generalized discharges of 3–6 Hz or faster on normal background. Brain magnetic resonances, cognitive and behavioral evaluation, and neurometabolic testing were performed to exclude symptomatic epilepsy. The patients have no or little cognitive impairment and neurodevelopmental comorbidities were included (Helbig, 2015).

The studies adhered to the guidelines of the International Committee of Medical Journal Editors with regard to patient consent for research or participation and received approval from the Ethics Committee of the Second Affiliated Hospital of Guangzhou Medical University (2021-hs-06).

### Trio-Based Whole-Exome Sequencing and Mutation Analysis

Blood samples of the probands and their biological parents were collected. Genomic DNA was extracted. Trio Whole Exome Sequencing (Trio-WES) was conducted as previously reported (Wang et al., 2018). Population-based filtration removed common variants presenting a minor allele frequency ≥ 0.005 in genome aggregation database (gnomAD). The potential disease-causing mutations were screened under five models, namely, epilepsy-associated gene model, dominant or *de novo* model, autosomal recessive inheritance model, X-linked model, and co-segregation analysis model. The candidate variants were validated by Sanger sequencing. Conservation of mutated positions was evaluated using sequence alignment of different species. All *GRIN2A* mutations were annotated based on the transcript NM_000833.4.

To evaluate the damaging effect of *GRIN2A* mutations, protein modeling was performed using Iterative Threading ASSEmbly Refinement (I-TASSER) software (Yang and Zhang, 2015; Zhang et al., 2017). The confidence of each modeling was quantitatively measured by C-score of -1.72. The three-dimensional structures were shown using PyMOL 1.7.

### cDNA Construction, Cell Culture, and Transfection

Rat GluN2A-K590, GluN2A-K879, and GluN2A-R1067 cDNA mutants were generated from the plasmid pcDNA3.1^+^-GluN2A by the site-directed mutagenesis kit, KOD-Plus-Neo (KOD-401, TOYOBO). As described in a previous study (Luo et al., 2002), the N-terminal GFP-tagged versions (GFP-GluN2A-K590N, GFP-GluN2A-K879R, and GFP-GluN2A-R1067W) were constructed using the GFP-GluN2A plasmid as a template. All these expression constructs were verified by DNA sequencing. Human embryonic kidney (HEK) 293 and 293T cells were grown in Dulbecco’s modified eagle medium (11995065, Gibco), supplemented with 10% fetal bovine serum (10099141, Gibco) and 1% penicillin and streptomycin (10378016, Gibco) in a humidified atmosphere of 5% CO_2_ at 37°C. Appropriate plasmids (2–4 μg per 35-mm dish) were transfected into the cells using the Lipofectamine 2000 Reagent (11668019, Invitrogen), according to the instructions of the manufacturer. To avoid NMDARs-mediated toxicity, 200 μM D, L-2-amino-5-phosphonovaleric acid (A8054, Sigma, United States) and 1 mM kynurenic acid (K3375, Sigma) were added to the culture medium. All experiments were performed in accordance with United Kingdom Animal Scientific Procedures Act (1986) following local ethical review (2021-hs-06).

### Whole-Cell Recordings and Immunofluorescence Analysis

Whole-cell current recordings were performed as previously described (the details listed in Supplementary Data 1) (Xu et al., 2018). Surface immunofluorescence staining has been described previously (Luo et al., 2002). Twenty-four hours after GluN1-1a/GFP-GluN2A or GluN1-1a/GFP-GluN2A-mutant cDNAs transfection, HEK293 cells were rinsed once with PBS, incubated with rabbit anti-GFP antibody (Chemicon) for 7 min subsequently. After rinsing three times, cells were incubated with secondary antibody (A11010, Invitrogen) for another 7 min. Cells were immediately fixed with 4% paraformaldehyde for 10 min following three washes. Images were acquired with a fluorescence microscope (BX51, Olympus) and analyzed using the MetaMorph image analysis software (Universal Imaging, West Chester, PA, United States). Red signal outlined around the transfected HEK 293 cells represented surface expression, and green signal represented intracellular expression.

### Cell Surface Biotinylation

HEK293T cells were incubated for 48 h after transfection and collected for extraction of total and surface protein. For total protein, cells were extracted using lysis buffer (FNN0021, Thermo Fisher Scientific), containing 1% phenylmethylsulfonyl fluoride and protease inhibitor cocktail (87786, Thermo Fisher Scientific). For surface protein, cells were permeabilized with permeabilization buffer (87786, Thermo Fisher Scientific, United States) supplemented with protease inhibitor cocktail (87786, Thermo Fisher Scientific). Surface protein was solubilized with solubilization buffer including protease inhibitor cocktail. The concentration of protein was measured using Bicinchoninic Acid (BCA) Protein Assay (23225, Thermo Fisher Scientific, United States). Equivalent amounts of the protein (200 μg for surface protein and 100 μg for total protein) was resolved over 7.5% sodium dodecyl sulfate-polyacrylamide gel electrophoresis, and transferred to polyvinylidene difluoride membrane (0.2 μm, 1620177, BIO-RAD, United States). The membranes were blocked within 5% non-fat milk for 2 h at room temperature and then incubated, respectively, with anti-GluN2A (1:4,000, ab124913, Abcam, United Kingdom), anti-β-actin (1:4,000, Proteintech, China), and anti-ATP1A1 (1:20,000, 14418-1-AP, Proteintech, China) antibodies at 4°C overnight. After washing the membranes in the mixture of tris-buffered saline and Tween 20 three times, the membranes were incubated with corresponding secondary antibodies for 2 h. Blots were representative of five independent experiments with similar results. Positive signals were analyzed by using ImageJ (National Institutes of Health, Bethesda, DC, United States).

### Analysis of Effect of *GRIN2A* Mutation and Phenotypic Variation

In an attempt to investigate the mechanism for phenotypic variation, epilepsy-related *GRIN2A* missense mutations and their corresponding phenotypes were systematically retrieved from the PubMed database using “*GRIN2A*” and “epilepsy” as search terms until December 2019. All *GRIN2A* mutations were annotated based on the transcript NM_000833.4. The functional alterations of the missense mutations were reviewed based on the results coming from two electrode voltage clamp recordings. The severity of functional changes was ranked based on the results of glutamate potency and response to Mg^2+^ block, and simultaneously referred to other electrophysiological evaluation indicators, such as current density, glycine potency, and protein expression, etc. The severity was classified into (1) severe functional alteration that was of equal to or more than five-fold increase or decrease of the glutamate potency and/or Mg^2+^ block or other minor functional changes, (2) intermediate functional alteration that was of more than two-fold and less than five-fold increase or decrease of the glutamate potency and/or Mg^2+^ block, and (3) mild functional alteration that was defined as less than or equal to two-fold increase or decrease of the glutamate potency and/or Mg^2+^ block in the mutations comparing to the wild type.

The phenotypes were divided into (1) severe phenotype, i.e., EE, (2) intermediate phenotype, including atypical benign partial epilepsy, Landau-Kleffner syndrome (LKS), continuous spikes and waves during slow sleep (CSWSS), myoclonic-astatic epilepsy, and focal epilepsy, and (3) mild phenotype that included benign epilepsy with centro-temporal spikes (BECTS) and IGEs.

### Statistical Analysis

All data values were expressed as mean ± SEM derived from at least three separate transfections. Graphpad Prism software and Statistical Package for the Social Sciences (SPSS) software were used for statistical analysis. The frequencies of *GRIN2A* variants in the cohort of IGEs and those in the general population were compared by two-sided Fisher’s exact test. Whole-cell current density and surface expression levels between wild-type and mutant receptors were compared by unpaired *t*-test. EC_50_ values between wild-type and mutant receptors (K590N, K879R, and R1067W) were compared by one-way ANOVA with Bonferroni *post hoc* multiple comparison test. The proportions of severe and mild phenotypes in different domains were compared by Pearson’s chi-square test. A *P* value of < 0.05 was considered to be statistically significant.

## Results

### Identification of *GRIN2A* Mutations

**FIGURE 1:**
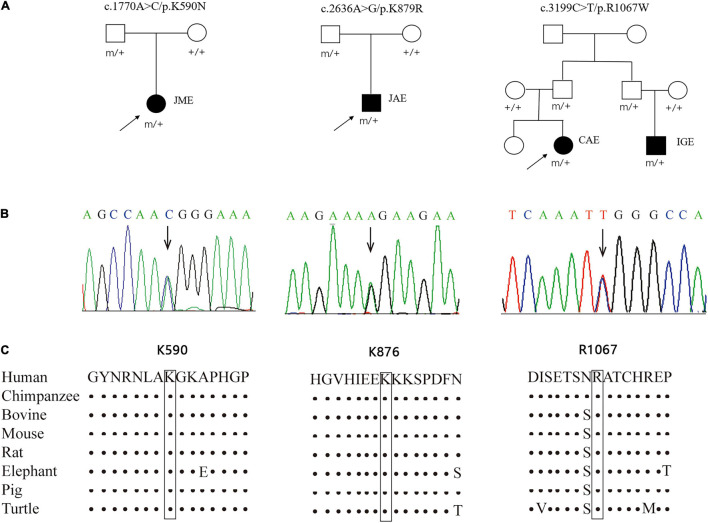
Genetic data about *GRIN2A* mutations. **(A)** Pedigrees of the three cases with *GRIN2A* mutations and their corresponding phenotypes. **(B)** DNA sequence chromatogram of the *GRIN2A* mutations. Arrows indicate the positions of the mutations. **(C)** The amino acid sequence alignment of the three mutations shows that residues K590, K879, and R1067 were highly conserved across vertebrates. JME, juvenile myoclonic epilepsy; JAE, juvenile absence epilepsy; CAE, childhood absence epilepsy; IGE, idiopathic generalized epilepsy.

Three novel inherited heterozygous missense *GRIN2A* mutations were identified in two unrelated sporadic cases and one family with IGEs (Figures 1A,B). Mutation c.1770A > C/p.K590N was identified in a case with JME, mutation c.2636A > G/p.K879R in a case with JAE, and mutation c.3199C > T/p.R1067W in two individuals in a family with CAE and unclassified IGE, respectively (Table 1). Mutations K590N and K879R presented at a minor allele frequency of 0.00002849 and 0.0002 in general population in gnomAD database, respectively, while mutation R1067W was not observed in gnomAD database. A statistical analysis showed that the frequency of the *GRIN2A* variants in the present cohort of IGEs was significantly higher than that in the general population or East-Asian population (in gnomAD) (3/176 vs. 67/282366 in general population, *p* = 0.000002, and 3/176 vs. 67/19946 in East-Asian population, *p* = 0.003625; Table 2). Mutations K590N, K879R, and R1067W were predicted to be damaging or probably damaging by 6, 14, and 18 out of the 25 *in silico* prediction tools, respectively (Supplementary Data 2). The amino acid sequence alignments showed that residues K590, K879, and R1067 were highly conserved across vertebrates (Figure 1C), indicating an important role of these residues in NMDARs functions. All cases had no other pathogenic or likely pathogenic mutations in genes known to be associated with seizure disorders.

**TABLE 1:** Clinical manifestations of the cases with *GRIN2A* mutations.

	**Case 1**	**Case 2**	**Case 3-1**	**Case 3-2**
Mutation	c.1770A>C/p.K590N	c.2636A>G/p.K879R	c.3199C>T/p.R1067W	c.3199C>T/p.R1067W
Phenotype	JME	JAE	CAE	MAE
Gender	Female	Male	Female	Male
Age (year)	20	27	10	8
Age of onset (year)	11	13	5	3
Seizure types	Myoclonic, GTCS	Absence, GTCS	Absence, GTCS	Myoclonic, atonic
Intelligence and development	Normal	Normal	Normal	Mild language and cognitive disabilities, autistic tendency
EEG findings	3-5 Hz generalized spike-waves and polyspike-waves	3 Hz generalized spike-waves and bi-frontal spike-waves	3 Hz generalized spike-waves and bi-centrotemporal spike-waves	2.5-3.5Hz generalized spike-waves and bi-frontocentral spike-waves
Brain MRI	Normal	Normal	Normal	Normal
Treatment	LTG	VPA, LTG	VPA	VPA, LTG
Prognosis	Seizure free	Seizure free	Seizure free	Seizure free
MAF in ExAC	0.00002849	0.0002	-	-

*JME, juvenile myoclonic epilepsy; JAE, juvenile absence epilepsy; CAE, childhood absence epilepsy; MAE, myoclonic-astatic epilepsy; GTCS, generalized tonic-clonic seizure; LTG, lamogrigine; VPA, valproic acid.*

**TABLE 2:** Gene-based burden analysis for *GRIN2A* mutations identified in this study.

	**Allele count/number in this study**	**Allele count/number in gnomAD-all populations**	**Allele count/number in gnomAD-East Asian populations**	**Allele count/number in controls of gnomAD-all populations**	**Allele count/number in controls of gnomAD-East Asian populations**
Identified *GRIN2A* mutations					
Chr16: 9927969: c.1770A>C/p.K590N	1/176 (0.00568)	10/282366 (0.00003542)	10/19950 (0.0005013)	6/120276 (0.00004989)	6/9960 (0.0006024)
Chr16: 9858765: c.2636A>G/p.K879R	1/176 (0.00568)	57/282532 (0.0002017)	57/19946 (0.002858)	27/120275 (0.0002245)	27/9956 (0.002712)
Chr16:9858202: c.3199C>T/p.R1067W	1/176 (0.00568)	–/–	–/–	–/–	–/–
**Total**	3/176 (0.01705)	67/282366 (0.00002373)	67/19946 (0.003359)	33/120275 (0.00002743)	33/9956 (0.003315)
***p* value**		0.000002	0.003625	0.000003	0.003766
**OR (95% CI)**		45.877–482.033	3.230–33.947	38.700–427.351	3.193-35.272

*p values and odds ratio were estimated with 2-sided Fisher’s exact test.*

*CI, confidence interval; gnomAD, Genome Aggregation Database; OR, odd ratio.*

**FIGURE 2:**
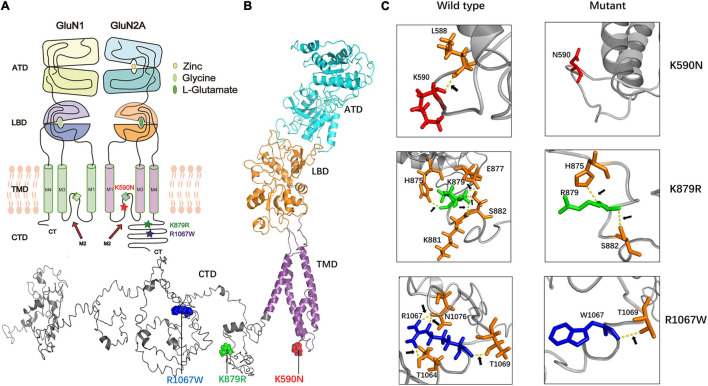
The alterations of hydrogen bonds with surrounding amino acids. **(A)** The locations of missense mutations in topological structure of GluN1/GluN2A. Residue K590N in GluN2A lies within the channel pore of the NMDAR, while residues K879R and R1067W lie in carboxyl-terminal domain. Mutation K590N was colored in red, mutation K876R was colored in green, and mutation R1067W was colored in blue. **(B)** Schematic illustration of the location of mutations in the three-dimensional structure of GluN2A. **(C)** Alterations of hydrogen bonds with surrounding amino acids. In wild type, residue K590 forms one hydrogen bond with L588. In the mutant, this hydrogen bond was destroyed. In wild type, residue K879 forms hydrogen bonds with H875, E877, K881, and S882 while in the mutant, the hydrogen bonds with E877 and K881 were destroyed. In wild type, residue R1067 forms hydrogen bonds with T1064, T1069, and N1076 while in the mutant, only hydrogen bond with T1069 was kept.

The mutation K590N was located in the intracellular domain and close to M2 domain, while the mutations K879R and R1067W were located in the carboxyl-terminal domain (CTD) (Figures 2A,B). The alterations of hydrogen bonds caused by the missense variants were further analyzed by protein modeling using Iterative Threading ASSEmbly Refinement (I-TASSER). Originally, residue K590 formed a hydrogen bond with residue L588. When lysine was replaced by asparagine at residue 590, the hydrogen bond was destroyed (Figure 2C). Residue K879 formed hydrogen bonds with residues H875, E877, K881, and S882, respectively. When lysine was replaced by arginine, the hydrogen bonds between residues E877 and K881 were broken, and the hydrogen bonds between H875 and S882 were preserved (Figure 2C). Residue R1067 formed two hydrogen bonds with N1076, and one hydrogen bond with T1064 and T1069 each. When arginine was replaced by tryptophan, the hydrogen bonds between N1076 and T1064 were destroyed, and only the hydrogen bond with T1069 were preserved (Figure 2C). The evidences indicated the mutations may alter the protein local conformation.

### Clinical Features of the Patients With *GRIN2A* Mutations

All affected cases showed childhood or adolescence-onset generalized epilepsy. Their clinical features were summarized in Table 1.

**FIGURE 3:**
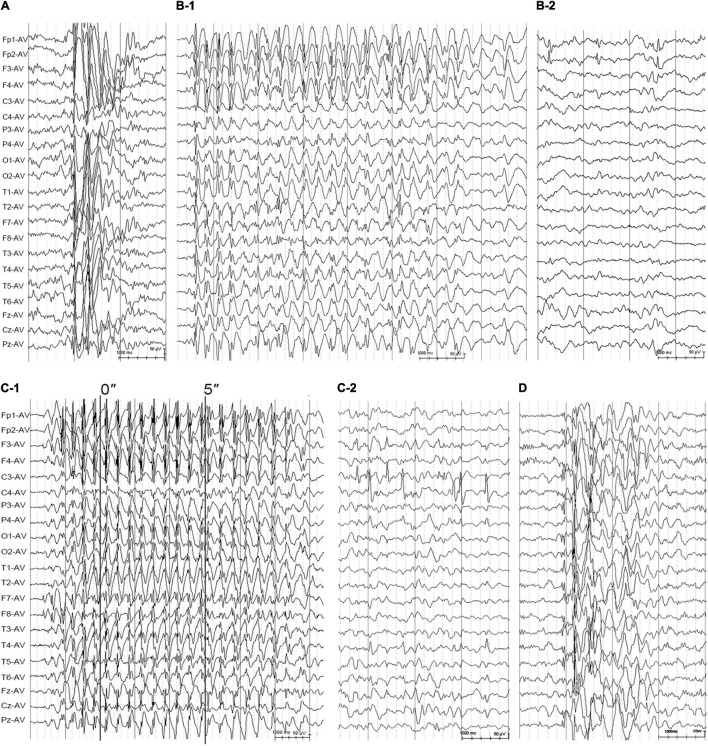
Electroencephalogram changes in the cases with idiopathic generalized epilepsy with *GRIN2A* mutations. **(A)** Interictal EEG for the patient with juvenile myoclonic epilepsy with mutation K590N obtained at the age of 14 years showed high-voltage generalized 3.5 Hz polyspike-and-waves. **(B)** Interictal EEG for the case with juvenile absence epilepsy with mutation K879R obtained at the age of 13 years showed 3–3.5 Hz spike- and -waves **(B-1)** and interictal bilateral frontal focal spikes **(B-2)**. **(C)** EEG for the patient with CAE with mutation R1067W obtained at the age of 5 years. The ictal EEG showed regular high voltage 3–3.5 Hz spike- and -waves with typical absence seizure **(C-1)**. The interictal EEG showed bilateral centro-temporal spikes during sleep **(C-2)**. **(D)** The interictal EEG of the patient with unclassified IGE with mutation R1067W obtained at the age of 3 years showed irregular spike-and-waves. EEG, electroencephalogram.

The patient with mutation K590N was a 20-year-old female with no family history of epilepsy and febrile seizures. She had the first generalized tonic-clonic seizure (GTCS) at the age of 11 years old. Thereafter, she had clusters of myoclonic jerks approximately 2–3 times per week. The video-EEG monitoring obtained at the age of 14 years old demonstrated intermittent high voltage 3–5 Hz generalized spike and polyspike-and-waves (Figure 3A). She was diagnosed as JME with myoclonic seizures and GTCSs, and was seizure free on lamotrigine 75 mg/day at 15 years old. The EEG obtained at the age of 15 years old showed that the epileptiform discharges dramatically decreased.

The patient with mutation K879R was a 27-year-old male with negative family history of epilepsy and febrile seizures. He had frequent absence seizures and one GTCS at the age of 13 years. The Video-EEG monitoring recorded frequently regular high voltage generalized 3 Hz spike-and-waves (Figure 3B-1) and occasionally bilateral frontal synchronous spike-and-waves (Figure 3B-2). He was diagnosed as JAE with typical absence seizures and GTCS. He was seizure free at 22 years old with the combination of valproate acid 500 mg/day, lamotrigine 200 mg/day, and levetiracetam 625 mg/day. The EEG returned to normal by 25 years old.

The family with mutation R1067W had two affected individuals. The proband was a 10-year-old girl, who was found to have repeated daily episodes of staring spells for about 10 s at 5 years old. The EEG obtained at 5 years old showed intermittent high voltage generalized 3 Hz spike-and-waves (Figure 3C-1) and bilateral independent centro-temporal spike-and-waves (Figure 3C-2). Frequent episodes of typical absence seizures were recorded. She was on valproate 18 mg/kg/day and seizure free for 5 years. The EEG obtained at 10 years old still showed epileptic discharges in left or right centro-temporal regions, but no focal seizure was found. The other patient was the proband’s cousin, an 8-year-old boy, appearing daily myoclonic and atonic seizures since he was 3 years old. He had mild speech delay, cognitive disabilities, and autistic tendencies. The EEG obtained at 3 years old showed high voltage irregular 2.5–3.5 Hz generalized polyspike-and-wave discharges (Figure 3D) and focal discharges in bilateral fronto-centro regions. He was diagnosed as unclassified IGE and was seizure free with the combination of valproate (25 mg/kg/day) and lamotrigine (3.5 mg/kg/day) by the age of six.

### Biophysiological Features of GluN2A-Mutants

**FIGURE 4:**
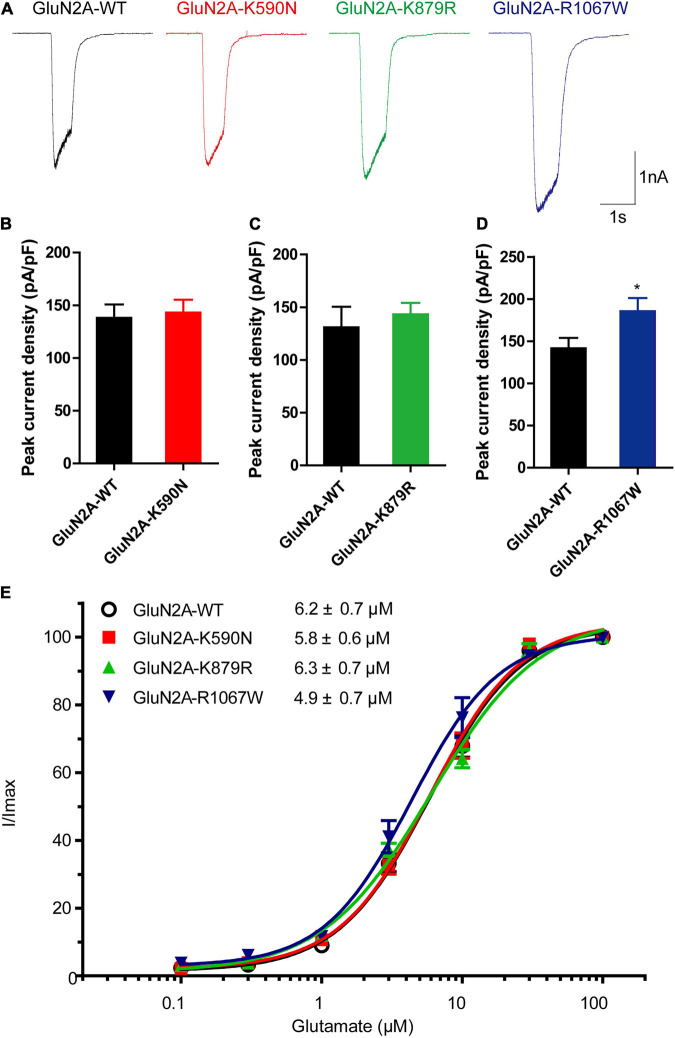
Electrophysiological functional alterations of GluN2A-mutant NMDARs. **(A)** Representative current traces of GluN1/GluN2A-WT, GluN2A-K590N, GluN2A-K879R, and GluN2A-R1067W NMDARs evoked by 20 μM glycine and 100 μM glutamate (current scale bar, 1 nA; time scale bar, 2 s). **(B)** Quantitative analysis of whole-cell current density of GluN2A-WT (*n* = 15) and GluN2A-K590N (*n* = 13) NMDARs (Student’s *t*-test, *p* > 0.05). **(C)** Quantitative analysis of whole-cell current density GluN2A-WT (*n* = 10) and GluN2A-K879R (*n* = 12) NMDARs (Student’s *t*-test, *p* > 0.05). **(D)** Quantitative analysis of whole-cell current density of GluN2A-WT (*n* = 12) and GluN2A-R1067W (*n* = 13) NMDARs (Student’s *t*-test, *p < 0.05). **(E)** Glutamate concentration-response curves of GluN1/GluN2A-WT (black open circles, *n* = 6), GluN2A-K590N (red squares, *n* = 6), GluN2A-K879R (green triangles, *n* = 6), and GluN2A-R1067W (blue triangles, *n* = 6) NMDARs (One-way ANOVA with Bonferroni *post hoc* multiple comparison test, *p* > 0.05). NMDAR, *N*-methyl-D-aspartate receptors.

To examine the functional changes of NMDARs caused by the mutants, electrophysiological experiments were conducted. As shown in Figures 4A,B, the average current density of GluN1/GluN2A-K590N NMDARs was similar to GluN1/GluN2A-WT NMDARs (143.1 ± 12.19 pA/pF, *n* = 13 vs. 138.1 ± 12.75 pA/pF, *n* = 15; *p* > 0.05). The average current density of GluN1/GluN2A-K879R NMDARs was slightly increased but not statistically significant from that of GluN1/GluN2A-WT NMDARs (143.4 ± 10.85 pA/pF, *n* = 12 vs. 130.9 ± 19.51 pA/pF, *n* = 10; *p* > 0.05; Figures 4A,C). However, the current density of GluN1/GluN2A-R1067W NMDARs was 31% higher than that of the wild type (185.6 ± 15.59 pA/pF, *n* = 13 vs. 141.6 ± 12.32 pA/pF, *n* = 12; *p* < 0.05; Figures 4A,D), suggesting a gain-of-function effect for GluN1/GluN2A-R1067W NMDARs.

To test whether the mutants change glutamate sensitivity of NMDARs, glutamate concentration-response assessments were performed. None of these mutants was revealed alteration in glutamate potency of NMDARs. The half-maximally effective concentration (EC50) was similar between GluN1/GluN2A-WT and mutant NMDARs (*n* = 6, *p* > 0.05; Figure 4E).

**FIGURE 5:**
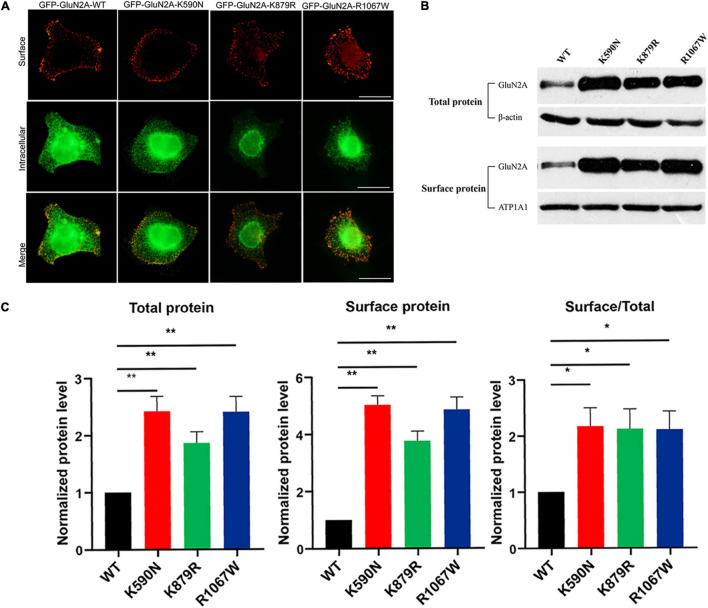
Surface and total expression of GluN2A-WT and GluN2A-mutant NMDARs detected by immunofluorescence staining and biotinylation. **(A)** Surface (red, upper row) and intracellular (green, lower row) expression of GluN1/GluN2A-WT or mutant NMDARs in HEK 293 cells. **(B)** Western blot detected the total and surface protein expression of GluN2A-WT, GluN2A-K590N, GluN2A-K879R, and GluN2A-R1067W NMDARs. **(C)** Quantitative analysis of the total and surface expression of GluN2A-WT and GluN2A-mutants NMDARs and their corresponding ratio of the surface/total expression as shown in panel **(B)** (*n* = 5. One-way ANOVA with Bonferroni *post hoc* multiple comparison test, *P < 0.05; **P < 0.01). NMDAR, *N*-methyl-D-aspartate receptors.

Immunofluorescence staining was performed to analyze the effect of mutants on cellular distribution of NMDARs. As shown in Figure 5A, all NMDARs with GluN2A mutants had abundant distribution in the membrane and cytoplasm as that of GluN1/GluN2A WT NMDARs. Biotinylation was conducted to assess the protein expression level. Compared to the wild type, both the total and surface expression levels of GluN2A-mutants NMDARs were significantly increased (Figures 5B,C; *p* < 0.01), and the ratios of surface and total expression of GluN2A-mutants were also higher than that of wild type (*p* < 0.05).

### Effect of *GRIN2A* Mutation and Phenotypic Variation

In order to understand the mechanism underlying phenotypic variations, all reported epilepsy-related *GRIN2A* missense mutations and their functional alterations were reviewed (Supplementary Data 3).

To date, 71 epilepsy-related missense mutations were reported. Electrophysiological tests were performed in 35 mutations previously (Endele et al., 2010; de Ligt et al., 2012; Carvill et al., 2013; DeVries and Patel, 2013; Lemke et al., 2013; Lesca et al., 2013; Conroy et al., 2014; Venkateswaran et al., 2014; Yuan et al., 2014; Fainberg et al., 2016; Retterer et al., 2016; Serraz et al., 2016; Singh et al., 2016; Swanger et al., 2016; Monies et al., 2017; von Stulpnagel et al., 2017; Dazzo et al., 2018; Hesse et al., 2018; Lindy et al., 2018; Lionel et al., 2018; Miao et al., 2018; Xu et al., 2018; Yang et al., 2018; Snoeijen-Schouwenaars et al., 2019; Strehlow et al., 2019). Among the 35 tested mutations, 10 mutations were demonstrated to cause gain of function (GOF), 16 mutations led to loss of function (LOF), and 9 mutations had no detectable electrophysiological changes in the aspects investigated.

Since the consequence of the mutations in the present study was GOF of NMDARs featured by increased current density and surface expression of protein, the correlation between GOF and phenotypic severity was analyzed. The detailed electrophysiological alterations and phenotypes were listed in Table 3 (Endele et al., 2010; Lemke et al., 2013; Yuan et al., 2014; Swanger et al., 2016; Chen et al., 2017; Ogden et al., 2017; Xu et al., 2018; Marwick et al., 2019a; Bertocchi et al., 2021). Three mutations (P552R, M817V, and L812M) significantly increased glutamate potency and glycine potency by over five-fold, and one mutation (N615K) led to a complete loss of Mg^2+^ blocker. The GOF of the four mutations were classified as severe, and the associated phenotypes were severe epilepsies, including two cases with early-onset epileptic encephalopathy and two with refractory epilepsy with severe developmental delay. Two mutations presented intermediate GOF. Mutation V452M caused 3.4-fold increase of glutamate potency and was associated with early infantile epileptic encephalopathy. Mutation K669N caused 3.1-fold increase of glutamate potency and was associated with intermediate phenotype CSWSS. Four mutations (N447K, V506A, P699S, and A243V) caused mild GOF, all of which were associated with mild phenotypes, including three cases with BECTS and one with unclassified epilepsy with incomplete penetrance. Three mutations identified in the study caused mild GOF or increased the expression of membrane protein, all of which were all associated with IGEs, which was classified as mild phenotype. This evidence indicated a quantitative correlation between the degree of GOF and the severity phenotype.

**TABLE 3:** Electrophysiological alterations featured by gain of function in *GRIN2A* mutations.

**No.**	**Mutation**	**Glutamate potency**	**Glycine potency**	**Current density**	**Mg^2+^ block**	**Ca^2+^ permeability**	**Zn^2+^ inhibition**	**Proton Sensitivity**	**Membrane expression**	**Function summary**	**Epileptic phenotypes**	**References**
1	c.1655C>G/p.P552R	↑↑↑(10-fold)	↑↑↑(20-fold)	+/–	NA	NA	NA	NA	NA	Severe GOF	Epilepsy & severe DD	Ogden et al., 2017
2	c.2449A>G/p.M817V	↑↑↑(9.5-fold)	↑↑↑(7.3-fold)	+/–	↓↓(3.33-fold)	NA	↓	↓	NA	Severe GOF	Refractory epilepsy & severe DD	Chen et al., 2017
3	c.2434C>A/p.L812M	↑↑↑(8-fold)	↑↑↑(8-fold)	+/–	↓(2-fold)	+/–	↓↓↓(loss)	↓	NA	Severe GOF	EOEE	Yuan et al., 2014
4	c.1845C>A/p.N615K	+/–	+/–	NA	↓↓↓(loss)	↓↓(2.88-fold)	NA	NA	NA	Severe GOF	EOEE	Endele et al., 2010; Marwick et al., 2019a; Bertocchi et al., 2021
5	c.1354G>A/p.V452M	↑↑(3.4-fold)	+/–	+/–	NA	NA	NA	NA	+/–	Intermediate GOF	EIEE	Swanger et al., 2016
6	c.2007G>T/p.K669N	↑↑(3.1-fold)	+/–	+/–	NA	NA	NA	NA	+/–	Intermediate GOF	CSWSS	Swanger et al., 2016
7	c.1341T>A/p.N447K	↑(2-fold)	NA	↑(1.2-fold)	↓(1.71-fold)	↑	↑	↑	+/–	Mild GOF	BECTS	Xu et al., 2018
8	c.1517T>C/p.V506A	↑(1.7-fold)	+/–	+/–	NA	NA	NA	NA	↑	Mild GOF	Unclassified Epilepsy, incomplete penetrance	Swanger et al., 2016
9	c.2095C>T/p.P699S	↑(1.6-fold)	+/–	+/–	NA	NA	NA	NA	↓	Mild GOF	BECTS	Swanger et al., 2016
10	c.728C>T/p.A243V	+/–	+/–	+/–	NA	NA	↓	+/–	+/–	Mild GOF	BECTS	Lemke et al., 2013
11	c.3199C>T/p.R1067W	+/–	NA	↑(1.3-fold)	NA	NA	NA	NA	↑↑	Mild GOF	CAE/MAE	This study
12	c.2636A>G/p.K879R	+/–	NA	+/–	NA	NA	NA	NA	↑↑	Mild GOF	JAE	This study
13	c.1770A>C/p.K590N	+/–	NA	+/–	NA	NA	NA	NA	↑↑	Mild GOF	JME	This study

*↑↑↑/ ↓↓↓: more than 5-fold increase/decrease compare to the wild type; ↑↑/↓↓: increase/decrease 2-5-fold compare to the wild type; ↑/↓: less or equal to 2- old increase/decrease compare to the wild type; +/–: no statistically significant difference between the mutant and the wild-type.*

*BECTS, benign epilepsy with centro-temporal spikes; DD, developmental delay; EOEE, early onset epileptic encephalopathy; EIEE, early infantile epileptic encephalopathy; CSWSS, continuous spike and waves during sleep; JME, juvenile myoclonic epilepsy; JAE, juvenile absence epilepsy; CAE, childhood absence epilepsy; MAE, myoclonic-astatic epilepsy; NA, not available.*

In previous studies, sixteen mutations were presented as LOF. Specifically, seven mutations were classified as severe featured by over five-fold decreased glutamate potency or complete trafficking defect (Supplementary Data 4; Swanger et al., 2016; Addis et al., 2017; Gao et al., 2017; Ogden et al., 2017; Strehlow et al., 2019), of which mutation V685G was associated with EE and the rest were associated with intermediate phenotypes. Four mutations were classified as intermediate LOF, and all associated with intermediate phenotypes. The remaining five mutations were ranked as mild LOF, of which three mutations (A727T, V734L, and R370W) were associated with BECTS. Thus, there was a tendency of correlation between the degree of LOF and phenotype severity. However, no definite conclusion could be drawn because majority of mutations with LOF were associated with intermediate phenotypes.

Nine mutations presented no detectable nor statistically significant alterations in the electrophysiological aspects examined (Supplementary Data 2).

There was no difference in phenotypic spectrum between the mutants with GOF and those with LOF. Electrophysiological alteration appeared not to be the only explanation for phenotypic variation. The previous study indicated that the molecular sub-regional location of mutations was associated with the damaging effects and, subsequently, the phenotypic severity (Tang et al., 2019). The relationship between the molecular sub-regional location of *GRIN2A* mutations and the severity of phenotype was therefore analyzed.

**FIGURE 6:**
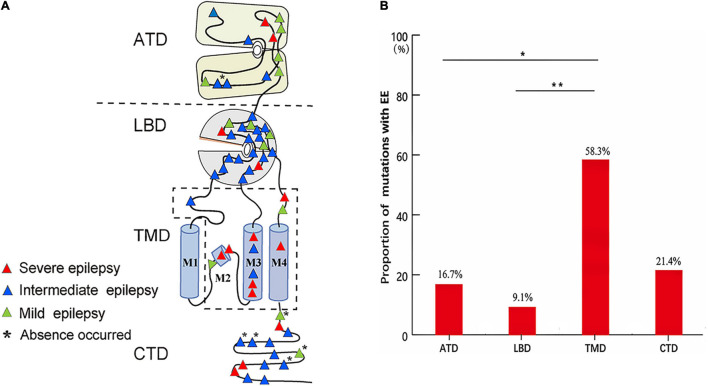
The locations and corresponding phenotypes of epilepsy-related missense *GRIN2A* mutations. **(A)** Topological distribution of the *GRIN2A* missense mutations with different phenotypes. **(B)** The proportion of *GRIN2A* mutations with EE in different molecular regions. The proportion of missense mutations with EE around TMD was significantly higher than that in ATD and LBD (Pearson’s chi-square test, *p < 0.05; **p < 0.01). ATD, amino terminal domain; LBD, ligand-binding domain; TMD, transmembrane domains; M1, M3, and M4, transmembrane domains; M2, re-entrant pore loop; CTD, carboxyl-terminal domain; EE, epileptic encephalopathy.

The epilepsy-related missense mutations were scattered over all domains of GluN2A except M1 helix (Figure 6A). The mutations located around the transmembrane domains (TMD) were more frequently associated with EE than those in amino- terminal domain (ATD) and ligand-binding domain (LBD) (Figure 6B), suggesting a molecular sub-regional effect. Previously, four patients with missense mutations had absence seizures, although they were diagnosed as LKS, CSWSS, and EE, respectively (Lemke et al., 2013; Lesca et al., 2013). Three of the four mutations were located in CTD. In this study, absence seizure-related mutations were also located in CTD. These data suggested a potential association between absence seizures and CTD.

## Discussion

The *GRIN2A* gene has been demonstrated to be associated with idiopathic focal epilepsy and EE. In the present study, three novel missense *GRIN2A* mutations were identified in unrelated cases with IGEs. These mutations presented significantly higher frequency in the case cohort than in general populations. Experimental studies demonstrated that these mutations caused mild GOF of NMDARs or expression alterations of GluN2A. Further analysis showed that the phenotypic severity was correlated with the degree of GOF and sub-regional locations. This study suggested that *GRIN2A* gene was potentially a candidate pathogenic gene of IGEs and would help understand the pathogenesis of IGEs.

The gene *GRIN2A* encodes GluN2A, a subunit of NMDARs, which are excitatory glutamate-gated channels with high Ca^2+^ permeability. *GRIN2A* is broadly expressed in multiple regions of the brain, including the cortex, cerebellum, and hippocampus since the embryonic period and is gradually increased during human development (Bar-Shira et al., 2015; Bagasrawala et al., 2017). A similar expression pattern was observed in rats (Sheng et al., 1994; Goebel and Poosch, 1999). GluN2A is critical for the formation and maturation of excitatory synapses and neuronal circuits (Swanger et al., 2016). To date, more than 140 *GRIN2A* mutations have been identified in focal epilepsy with or without speech disorders and EE. In the present study, three missense *GRIN2A* mutations were identified in the patients with IGEs. These mutations presented no or low allele frequencies in the gnomAD database and statistically higher frequency in the cohort of IGEs than in the populations of gnomAD. Experimental studies revealed increased current density in mutant NMDARs with R1067W and increased membrane protein expression in the three mutants. Considering that NMDARs generally mediate excitatory neurotransmission and are critical for the regulation of neuronal excitability in the brain, it potentially explains the association between *GRIN2A* variants and epilepsy. This study provided an insight into the underlying mechanism for the pathogenesis of IGEs.

Previously, 10 *GRIN2A* mutations with epilepsy were identified as GOF through two-electrode voltage clamp recordings (Endele et al., 2010; Lemke et al., 2013; Yuan et al., 2014; Swanger et al., 2016; Chen et al., 2017; Ogden et al., 2017; Xu et al., 2018). Further analysis in the study revealed that the severe GOF were associated with EE, while the mild GOF were mainly associated with mild phenotypes, specifically, BECTS or IGEs, indicating a quantitative correlation between functional alteration and phenotypic severity. In the present study, mutation R1067W had no allele frequency in the general population, caused more severe functional changes, and was predicted to be damaging by more *in silico* tools than mutations K590N and K879R. Clinically, mutation R1067W was associated with relatively more severe phenotypes, i.e., unclassified IGE with earlier onset, more frequent seizures, poorer response to AEDS, more than one individual affected, and potentially intellectual and developmental impairments. These findings provided additional pieces of evidence on quantitative correlation between functional alteration and phenotypic severity, potentially explaining the mild clinical manifestation, and incomplete penetrance.

In previous studies, incomplete penetrance and intra or inter-familial phenotypic variability of *GRIN2A* mutations were commonly observed in the families with idiopathic focal epilepsy (Lemke et al., 2013; Lesca et al., 2013). The phenomenon was also observed in the present study. The underlying mechanism remains undetermined. The numerous genomic variations in each individual and environmental factors might modify the phenotype. Generally, the variants with strong pathogenicity usually produce a relatively accordant phenotype, such as the variants in genes related to epileptic encephalopathy. In contrast, the variants with less pathogenicity tend to present phenotypic variation and incomplete penetrance and be associated with the mild functional alteration. In this study, the mutations K590N and K879R presented low frequency in control populations and lead to milder alterations of GluN2A expression, which might be one of the explanations for incomplete penetrance and intra or inter-familial phenotypic variability. It is possible that the *GRIN2A* variants with the mild impact played a risk, rather than a causal role in IGE, and were associated with only increased susceptibility to epilepsy.

Generally, the electrophysiological properties of channels are directly related to neuronal excitability, which determines the susceptibility of epilepsy. However, mutations with electrophysiological LOF of NMDARs have been identified previously (Swanger et al., 2016; Addis et al., 2017; Gao et al., 2017; Ogden et al., 2017; Strehlow et al., 2019). Truncating mutations and gross deletions of *GRIN2A* gene were also reported (Endele et al., 2010; Lesca et al., 2013). In animal models, homozygotes of targeted null *GRIN2A* exhibited jumpiness, increased locomotor activity, and loss of analgesic tolerance after repeated morphine doses (Sakimura et al., 1995; Kadotani et al., 1996). In *Grin2a* knockout mice, spontaneous epileptiform discharges were detected (Salmi et al., 2018, 2019). These clues indicated that loss of GluN2A protein was associated with increased neuronal excitability. Therefore, LOF of *GRIN2A* was potentially pathogenic for epilepsy. The analysis revealed a tendency of quantitative correlation between the degree of LOF and phenotypic severity. However, a conclusion cannot be drawn for LOF at present due to the data limitation. Additionally, several mutations had no detectable electrophysiological alterations. The pathogenic mechanism for these mutations was unknown. Electrophysiological alterations of NMDARs appeared to be not the only explanation of epileptogenesis. *GRIN2A* is broadly expressed in the human brain since the embryonic period, indicating *GRIN2A* potentially plays a role in neurodevelopment. Clinically, patients with *GRIN2A* mutations had variable neurodevelopmental abnormalities. It is, therefore, possible that *GRIN2A* mutations will cause neurodevelopmental abnormalities and subsequently secondary epilepsy, for which the underlying mechanism warrants further studies.

A recent study showed that molecular sub-regional locations of mutations were associated with the pathogenicity (Tang et al., 2019). Previous studies showed that the locations of missense mutations affected the severity of developmental phenotypes. The missense mutations in transmembrane and linker domains were associated with severe developmental delay (Strehlow et al., 2019). The present study revealed that the severity of epileptic phenotypes was also associated with the locations of missense mutations. Particularly, the missense mutations in TMD of GluN2A were more frequently associated with more severe phenotypes of epilepsy, whereas the mutations in ATD and LBD were more frequently associated with milder epilepsies. Additionally, five of the six absence associated mutations were located in CTD. These findings suggest the phenotypes were affected by a molecular sub-regional effect of *GRIN2A* mutations.

In the present study, two variants K879R and R1067W were located in C-terminal. C-terminal is less conservative in evolution with divergence among different species (Hedegaard et al., 2012). However, K879 and R1067 and their interacting residues were conservative residues (Figures 1C, 2C). Variant R1067W had no allele frequency in controls of general population, and led to functional alterations of NMDARs. In previous studies, thirteen C-term variants with no or low allele frequency in general population were reported in the patients with epilepsy, even in the patients with epileptic encephalopathy. Functional studies had been performed in two C-term variants previously (Addis et al., 2017; Mota Vieira et al., 2020). The variant of GluN2A-N976S had no detectable electrophysiological alteration, while GluN2A-S1459G was proved to reduce spontaneous miniature excitatory synaptic current (mEPSC) frequency, decrease NMDAR surface expression, disrupt NMDAR interactions, and reduce synaptic function (Mota Vieira et al., 2020). It was suggested that the pathogenicity of C-term variants was variable and some of variants were potentially pathogenic, for which further experimental investigations were needed.

Clinically, features of BECTS and IGEs might consecutively or contemporarily coexist in the same patients (Esmail et al., 2016; Verrotti et al., 2017). In the present study, focal discharges were also observed in the patients with JAE and CAE (Figure 3). Both BECTS and IGEs were associated with *GRIN2A* mutations, which were potentially the common genetic basis of the two phenotypes. However, the absence-associated mutations were mainly located in CTD, while BECTS-associated mutations did not appear in this region. The difference in the distribution of mutations would potentially explain the phenotypic variation, for which the underlying mechanisms warrants further investigation.

This study has several limitations. More cases with IGEs are required to confirm the association between *GRIN2A* variants and IGEs. Further studies should be performed to elucidate the mechanism underlying the pathogenesis of IGEs. Recently, increasing evidence showed that triheteromers (GluN1/2A/2B) were prominent in the alterations of electrophysiological functions as compared with diheteromers (GluN1/2A) (Marwick et al., 2019b), which is potentially an alternative approach to determine the electrophysiological functional alterations of *GRIN2A* variants.

In conclusion, the present study revealed *GRIN2A* gene was potentially a candidate pathogenic gene of IGEs. The molecular sub-regional effects of missense mutations and the quantitative correlation between the degree of GOF and the phenotypic severity provided evidence to explain the relatively mild clinical phenotypes and incomplete penetrance of *GRIN2A* variants, which would help understand the underlying mechanisms of phenotypic variation.

## Data Availability Statement

The datasets presented in this study can be found in online repositories. The names of the repository/repositories and accession number(s) can be found in the article/Supplementary Material.

## Ethics Statement

The studies involving human participants were reviewed and approved by the Ethics Committee of the Second Affiliated Hospital of Guangzhou Medical University (2021-hs-06). Written informed consent to participate in this study was provided by the participants’ legal guardian/next of kin.

## Author Contributions

X-RL, J-HL, and W-PL designed and conceptualized the study, analyzed and interpreted the data, and drafted and revised the manuscript. J-HL and W-PL had full access to all of the data in the study and take responsibility for the integrity of the data and the accuracy of the data analysis. X-RL, B-ML, and Y-HY contributed to the clinical data collection. X-RL, X-XX, S-ML, and C-YF contributed to the electrophysiological and protein expression analysis. X-RL, T-TY, BT, Y-WS, and TS contributed to *in silico* analysis and the statistical analysis. All authors collected the data and revised and contributed to wrote the manuscript.

## Conflict of Interest

The authors declare that the research was conducted in the absence of any commercial or financial relationships that could be construed as a potential conflict of interest.

## Publisher’s Note

All claims expressed in this article are solely those of the authors and do not necessarily represent those of their affiliated organizations, or those of the publisher, the editors and the reviewers. Any product that may be evaluated in this article, or claim that may be made by its manufacturer, is not guaranteed or endorsed by the publisher.

## Footnotes

1
https://www.proteinatlas.org/ENSG00000183454-GRIN2A/tissue

## Abbreviations

NMDAR: *N*-methyl-D-aspartate receptor
ATD: amino-terminal domain
LBD: ligand-binding domain
TMD: transmembrane domains
CTD: carboxyl-terminal domain
LOF: loss of function
GOF: gain of function
GTCS: generalized tonic-clonic seizure
IGE: idiopathic generalized epilepsy
JME: juvenile myoclonic epilepsy
JAE: juvenile absence epilepsy
CAE: childhood absence epilepsy
EGTCS: epilepsy with generalized tonic-clonic seizures alone
BECTS: benign epilepsy with centro-temporal spikes
LKS: Landau-Kleffner syndrome
CSWSS: continuous spikes and waves during slow sleep
EE: epileptic encephalopathy.
