# Supplementary material for: GRIN2A Variants Associated With Idiopathic Generalized Epilepsies
Source: Front Mol Neurosci. 2021 Oct 14;14:720984. doi: 10.3389/fnmol.2021.720984 (PMC8551482; doi:10.3389/fnmol.2021.720984)
Supplement: Supplementary file 1 [file Table_1.docx]

**Supplementary data 1**

**Whole-cell recordings**

Whole-cell current recordings were performed as previously described [Xu XX, et al. Neurosci Bull, 2018, PMID: 28936771]. Briefly, cDNA constructs encoding EGFP, GluN1-1a, and wild-type GluN2A (hereafter referred as GluN2A-WT) or mutant GluN2A were co-transfected into HEK 239T cells. 24 hours after transfection, the EGFP-positive cells were patch-clamped, and the electrodes with resistance of 3-5 MΩ were used. The extracellular solution was composed of 135 mM NaCl, 5 mM KCl, 2 mM CaCl_2_, 10 mM HEPES at pH 7.4 with NaOH and the Osmolarity was adjusted to 300 mOsms with sucrose. The internal solution filled in the electrodes was 140 mM CsCl, 2 mM MgATP, 10 mM EGTA, 10 mM HEPES at pH 7.3 with CsOH and the osmolality was about 300-310 mOsms. Drugs were applied to the lifted cells using an RSC-160 system (Biologic Science Instruments) with a continuous perfusion of extracellular solution in the recording chamber. For the glutamate (0.1-100 μM) concentration-response curves, the percentage current was normalized to the current value obtained at 300 μM glutamate and 20μM glycine. HEK 293T cells were all clamped at -60 mV. All the reagents were purchased from Sigma. Off-line data analysis were performed with Clampfit 9 (Axon Instruments).
