# Supplementary material for: GRIN2A Variants Associated With Idiopathic Generalized Epilepsies
Source: Front Mol Neurosci. 2021 Oct 14;14:720984. doi: 10.3389/fnmol.2021.720984 (PMC8551482; doi:10.3389/fnmol.2021.720984)
Supplement: Supplementary file 2 [file Table_2.docx]

| **Supplementary data 2. Predictions for the pathogenicity by the in silico programs** | | | |
| --- | --- | --- | --- |
|  | **c.1770A>C/p.K590N** | **c.2636A>G/p.K879R** | **c.3199C>T/****p.R1067W** |
| **Damage predicting (25 predicting tools)** | 6 | 14 | 18 |
| **SIFT** | Tolerable (0.504) | Tolerable (0.902) | Damaging (0.001) |
| **Polyphen-2_HDIV** | Benign (0.053) | Probably damaging (0.997) | Probably_damaging (1.0) |
| **LRT** | Deleterious (0.001) | Deleterious (0.000) | Deleterious (0.000) |
| **MutationTaster** | Disease causing (0.992) | Disease-causing (1.000) | Disease_causing (1.000) |
| **Mutation Assessor** | Low (0.825) | Low (1.93) | Medium (2.485) |
| **FATHMM** | Tolerable (0.83) | Tolerable (2.78) | Tolerable (2.52) |
| **PROVEAN** | Tolerable (0.44) | Tolerable (-0.11) | Tolerable (-2.45) |
| **VEST3** | Tolerable (0.053) | Damaging (0.635) | Damaging (0.92) |
| **MetaSVM** | Tolerable (-1.051) | Tolerable (-1.176 ) | Tolerable (-1.096) |
| **MetaLR** | Tolerable (0.073) | Tolerable (0.046) | Tolerable (0.094) |
| **M-CAP** | Damaging (0.184) | Damaging (0.145) | Damaging (0.536) |
| **CADD** | Tolerable (18.62) | Damaging (20.2) | Damaging (25.7) |
| **DANN** | Tolerable (0.989) | Tolerable (0.823) | Damaging (0.999) |
| **FATHMM_MKL** | Damaging (0.689) | Damaging (0.962) | Damaging (0.890) |
| **Eigen** | Tolerable (-0.144) | Damaging (0.450) | Damaging (0.397) |
| **GenoCanyon** | Tolerable (0.07) | Damaging (1.000) | Tolerable (0.784) |
| **fitCons** | Tolerable (0.554) | Tolerable (0.615) | Tolerable (0.554) |
| **GERP++** | Conserved (4.94) | Conserved (5.52) | Conserved (4.34) |
| **phyloP** | Nonconserved (0.052) | Conserved (5.858) | Conserved (2.055) |
| **phastCons** | Nonconserved (0.895) | Conserved (1.000) | Conserved (1.000) |
| **SiPhy** | Nonconserved (10.292) | Conserved (14.858) | Conserved (12.891) |
| **REVEL** | Tolerable (0.117) | Tolerable (0.175) | Tolerable (0.299) |
| **ReVe** | Tolerable (0.154) | Tolerable (0.461) | Damaging (0.792) |
| **ClinPred** | Benign (0.07774137) | Benign (0.14147237) | Pathogenic (0.97702652) |
| **SNAP** | Neutral (-31) | Neutral (-71) | Effect (35) |
