# Supplementary material for: GRIN2A Variants Associated With Idiopathic Generalized Epilepsies
Source: Front Mol Neurosci. 2021 Oct 14;14:720984. doi: 10.3389/fnmol.2021.720984 (PMC8551482; doi:10.3389/fnmol.2021.720984)
Supplement: Supplementary file 3 [file Table_3.docx]

**Supplementary data 3. Phenotypes and electrophysiological functions of epilepsy-related *GRIN2A* missense mutations**

| **No.** | **Mutations** | **Locations** | **Allele count in gnomAD-all populations** | **Allele count in gnomAD-East Asian populations** | **Epileptic syndrome** | **DD** | **Phenotype severity** | **Functional alteration**  **(glutamate potency)** | **References** |
| --- | --- | --- | --- | --- | --- | --- | --- | --- | --- |
| 1. | c.2T>C/p.M1T | ATD | - | - | LKS | + | Intermediate |  | (Carvill et al., 2013) |
| 2. | c.236C>G/p.P79R | ATD | 0.000003986 | 0.000009141 | CSWSS | + | Intermediate | LOF (3.5-fold) | (Lemke et al., 2013); (Serraz et al., 2016) |
| 3. | c.547T>A/p.F183I | ATD | 0.00004386 | 0.00005486 | BECTS | + | Mild | No change | (Lemke et al., 2013); (Serraz et al., 2016) |
| 4. | c.551T>G/p.I184S | ATD | - | - | CSWSS | + | Intermediate | No change | (Lesca et al., 2013); (Serraz et al., 2016) |
| 5. | c.691T>C/p.C231R | ATD | - | - | ABPE | NA | Intermediate |  | (Yang et al., 2018) |
| 6. | c.692G>A/p.C231Y | ATD | - | - | LKS | + | Intermediate | LOF (4.5-fold) | (Lemke et al., 2013); (Serraz et al., 2016) |
| 7. | c.728C>T/p.A243V | ATD | - | - | BECTS | NA | Mild | GOF (Zn^2+^ inhibition decreased) | (Lemke et al., 2013) |
| 8. | c.869C>T/p.A290V | ATD | 0.00003543 | 0.00001829 | BECTS | - | Mild | No change | (Lemke et al., 2013); (Serraz et al., 2016) |
| 9. | c.883G>A/p.G295S | ATD | 0.00004784 | 0.00004572 | BECTS | - | Mild | No change | (Lesca et al., 2013); (Serraz et al., 2016) |
| 10. | c.905C>T/p.A302V | ATD | - | - | EE | + | Severe |  | (Monies et al., 2017) |
| 11. | c.989C>T/p.P330L | ATD | 0.00003551 | 0.00003661 | EE | + | Severe |  | (von Stulpnagel et al., 2017) |
| 12. | c.1108C>T/p.R370W | ATD | 0.000003980 | - | BECTS | NA | Mild | LOF (Zn^2+^ inhibition increased) | (Lemke et al., 2013); (Serraz et al., 2016) |
| 13. | c.1232T>A/p. L411Q | S1 | - | - | Unclassified epilepsy | + | NA |  | (Strehlow et al., 2019) |
| 14. | c.1306T>C/p.C436R | S1 | - | - | ABPE | + | Intermediate | LOF (trafficking damage) | (Lemke et al., 2013); (Addis et al., 2017); (Serraz et al., 2016) |
| 15. | c.1341T>A/p.N447K | S1 | 0.00004786 | 0.00006402 | BECTS | NA | Mild | GOF (2-fold) | (Xu et al., 2018) |
| 16. | c.1354G>A/p.V452M | S1 | 0.0003222 | 0.0003077 | EIEE | NA | Severe | GOF (3.4-fold) | (Singh et al., 2016) |
| 17. | c.1364G>A/p.C455Y | S1 | - | - | ABPE | NA | Intermediate |  | (Yang et al., 2018) |
| 18. | c.1447G>A/p.G483R | S1 | - | - | CSWSS/ABPE | + | Intermediate | LOF (26.9-fold) | (Lesca et al., 2013); (Swanger et al., 2016) |
| 19. | c.1492G>A/p.G498S | S1 | - | - | CSWSS | + | Intermediate |  | (Strehlow et al., 2019) |
| 20. | c.1493G>A/p.G498D | S1 | - | - | Unclassified epilepsy | + | NA |  | (von Stulpnagel et al., 2017) |
| 21. | c.1510C>T/p.R504W | S1 | 0.00003443 | - | CSWSS | + | Intermediate | No change | (Lesca et al., 2013); (Swanger et al., 2016) |
| 22. | c.1517T>C/p.V506A | S1 | - | - | Unclassified epilepsy | + | NA | GOF (1.7-fold) | (DeVries and Patel, 2013); (Swanger et al., 2016) |
| 23. | c.1552C>T/p.R518C | S1 | - | - | LKS | + | Intermediate |  | (Lindy et al., 2018); (Strehlow et al., 2019) |
| 24. | c.1553G>A/p.R518H | S1 | - | - | LKS/CSWSS/ABPE | + | Intermediate | LOF (complete) | (Lesca et al., 2013); (Swanger et al., 2016) |
| 25. | c.1580C>G/p.P527R | S1 | - | - | Unclassified epilepsy | + | NA |  | (Lindy et al., 2018) |
| 26. | c.1592C>T/p.T531M | S1 | - | - | CSWSS | + | Intermediate | LOF (complete) | (Carvill et al., 2013); (Strehlow et al., 2019) |
| 27. | c.1642G>A/p.A548T | S1-M1 | - | - | LKS | + | Intermediate | LOF (4.1-fold) | (Lesca et al., 2013); (Ogden et al., 2017) |
| 28. | c.1655C>G/p.P552R | S1-M1 | - | - | Unclassified epilepsy | + | NA | GOF (10-fold) | (de Ligt et al., 2012); (Ogden et al., 2017) |
| 29. | c.1832T>A/p.L611Q | M2 | - | - | Unclassified epilepsy | + | NA |  | (Strehlow et al., 2019) |
| 30. | c.1841A>G/p.N614S | M2 | - | - | EE | + | Severe |  | (von Stulpnagel et al., 2017) |
| 31. | c.1845C>A/p.N615K | M2 | - | - | EOEE | + | Severe | GOF (loss of Mg^2+^ block) | (Endele et al., 2010); (Marwick et al., 2019); (Bertocchi et al., 2021) |
| 32. | c.1903G>A/p.A635T | M3 | - | - | EE | + | Severe |  | (Strehlow et al., 2019) |
| 33. | c.1930A>G/p.S644G | M3 | - | - | Unclassified epilepsy | + | NA |  | (Lindy et al., 2018) |
| 34. | c.1936A>G/p.T646A | M3 | - | - | EE | NA | Severe |  | (von Stulpnagel et al., 2017) |
| 35. | c.1943A>G/p.N648S | M3 | - | - | Unclassified epilepsy | NA | NA |  | (Retterer et al., 2016); (Strehlow et al., 2019) |
| 36. | c.1945C>G/p.L649V | M3 | - | - | Unclassified epilepsy | + | NA |  | (de Ligt et al., 2012); (Strehlow et al., 2019) |
| 37. | c.1954T>G/p.F652V | M3 | - | - | CSWSS | + | Intermediate | GOF (single channel) | (Lesca et al., 2013) |
| 38. | c.1959G>A/p.M653I | M3 | - | - | Unclassified epilepsy | + | NA |  | (Strehlow et al., 2019) |
| 39. | c.1957A>G/p.M653V | M3 | - | - | Focal epilepsy | + | Intermediate |  | (Strehlow et al., 2019) |
| 40. | c.1961T>C/p.I654T | M3 | - | - | EE | + | Severe |  | (Strehlow et al., 2019) |
| 41. | c.2007G>T/p.K669N | S2 | - | - | CSWSS | + | Intermediate | GOF (3.1-fold) | (Lesca et al., 2013); (Swanger et al., 2016) |
| 42. | c.2050A>G/p.T684A | S2 | - | - | Focal epilepsy | + | Intermediate |  | (Lionel et al., 2018); (Strehlow et al., 2019) |
| 43. | c.2054T>G/p.V685G | S2 | - | - | EE | + | Severe | LOF (79-fold) | (Swanger et al., 2016) |
| 44. | c.2081T>C/p.I694T | S2 | - | - | LKS | + | Intermediate | LOF (2.9-fold) | (Lesca et al., 2013); (Swanger et al., 2016) |
| 45. | c.2095C>T/p.P699S | S2 | - | - | BECTS | - | Mild | GOF (1.6-fold) | (Lemke et al., 2013); (Swanger et al., 2016) |
| 46. | c.2113A>G/p.M705V | S2 | - | - | ABPE | + | Intermediate | LOF (2.1-fold) | (Lemke et al., 2013); (Swanger et al., 2016); (Addis et al., 2017) |
| 47. | c.2138T>G/p.V713G | S2 | - | - | Unclassified epilepsy | + | NA |  | (Strehlow et al., 2019) |
| 48. | c.2140G>A/p.E714K | S2 | - | - | CSWSS | + | Intermediate | No change | (Lemke et al., 2013); (Addis et al., 2017) |
| 49. | c.2146G>A/p.A716T | S2 | - | - | ABPE/BECTS | + | Intermediate | LOF (5.9-fold) | (Fainberg et al., 2016); (Lesca et al., 2013); (Swanger et al., 2016) |
| 50. | c.2179G>A/p.A727T | S2 | - | - | BECTS | - | Mild | LOF (1.5-fold) | (Lemke et al., 2013); (Swanger et al., 2016) |
| 51. | c.2191G>A/p.D731N | S2 | - | - | ABPE | + | Intermediate | LOF (complete) | (Lesca et al., 2013); (Gao et al., 2017); (Swanger et al., 2016) |
| 52. | c.2197G>A/p.A733T | S2 | - | - | Unclassified epilepsy | + | NA |  | (Lindy et al., 2018) |
| 53. | c.2200G>C/p.V734L | S2 | - | - | BECTS | - | Mild | LOF (1.5-fold) | (Lemke et al., 2013); (Swanger et al., 2016) |
| 54. | c.2314A>G/p.K772E | S2 | - | - | ABPE | + | Intermediate | LOF (1.4-fold) | (Lemke et al., 2013); (Swanger et al., 2016) |
| 55. | c.2434C>A/p.L812M | S2-M4 | - | - | EOEE | + | Severe | GOF (8-fold) | (Yuan et al., 2014); (Chen et al., 2017) |
| 56. | c.2441T>C/p.I814T | S2-M4 | 0.00001592 | 0.000009141 | BECTS | NA | Mild | No change | (Lemke et al., 2013); (Addis et al., 2017) |
| 57. | c.2449A>G/p.M817V | M4 | - | - | EE | + | Severe | GOF (9.5-fold) | (Venkateswaran et al., 2014) (Chen et al., 2017) |
| 58. | c.2453C>A/p.A818E | M4 | - | - | Unclassified epilepsy | + | NA |  | (Strehlow et al., 2019) |
| 59. | c.2663C>T/p.T888M | CTD | 0.00003537 | 0.00002494 | EE | + | Severe |  | (Hesse et al., 2018) |
| 60. | c.2710A>T/p.I904F | CTD | - | - | ABPE | + | Intermediate |  | (Lemke et al., 2013) |
| 61. | c.2723C>T/p.S908F | CTD | - | - | Focal epilepsy | NA | Intermediate |  | (Dazzo et al., 2018) |
| 62. | c.2797G>A/p.D933N | CTD | - | - | LKS | + | Intermediate | No change | (Lesca et al., 2013); (Addis et al., 2017) |
| 63. | c.2927A>G/p.N976S | CTD | 0.000007959 | 0.000009145 | ABPE/ CSWSS | - | Intermediate | No change | (Lemke et al., 2013); (Addis et al., 2017) |
| 64. | c.3073T>A/p. S1025T | CTD | - | - | MAE | NA | Mild |  | (von Stulpnagel et al., 2017) |
| 65. | c.3751G>A/p.D1251N | CTD | - | - | ABPE/Absence epilepsy | + | Intermediate |  | (Lesca et al., 2013) |
| 66. | c.3827C>G/p.A1276G | CTD | 0.0005730 | 0.0005157 | BECTS/CSWSS | NA | Intermediate |  | (Lesca et al., 2013) |
| 67. | c.3952C>T/p. R1318W | CTD | 0.00001994 | 0.000009185 | EE | NA | Severe |  | (von Stulpnagel et al., 2017) |
| 68. | c.4126C>A/p. R1376S | CTD | - | - | Unclassified epilepsy | NA | NA |  | (von Stulpnagel et al., 2017) |
| 69. | c.4139G>C/p. G1380A | CTD | - | - | EE | + | Severe |  | (Miao et al., 2018) |
| 70. | c.4153G>T/p.D1385Y | CTD | - | - | ABPE | NA | Intermediate |  | (Yang et al., 2018) |
| 71. | c.4355G>A/p.R1452H | CTD | 0.00001593 | 0.00001829 | Focal epilepsy | + | Mild |  | (Snoeijen-Schouwenaars et al., 2019) |

**Abbreviations:** ATD: amino-terminal domain; LBD: ligand-binding domain; TMD: transmembranedomains; CTD: carboxyl-terminal domain; DD: developmental delay; ABPE: atypical benign partial epilepsy; LKS: Landau-Kleffner syndrome; CSWSS: continuous spikes and waves during slow sleep; BECTS: benign epilepsy with centro-temporal spikes; MAE: myoclonic-atastic epilepsy; EE: epileptic encephalopathy; EOEE: early onset epileptic encephalopathy; EIEE: early infantile epileptic encephalopathy; GOF: gain of function; LOF: loss of function; NA: not available.

**References**

Addis, L., Virdee, J.K., Vidler, L.R., Collier, D.A., Pal, D.K., and Ursu, D. (2017). Epilepsy-associated GRIN2A mutations reduce NMDA receptor trafficking and agonist potency - molecular profiling and functional rescue. *Sci Rep.* 7: 66. doi: 10.1038/s41598-017-00115-w.

Bertocchi, I., Eltokhi, A., Rozov, A., Chi, V.N., Jensen, V., Bus, T., et al. (2021). Voltage-independent GluN2A-type NMDA receptor Ca(2+) signaling promotes audiogenic seizures, attentional and cognitive deficits in mice. *Commun Biol.* 4: 59. doi: 10.1038/s42003-020-01538-4.

Carvill, G.L., Regan, B.M., Yendle, S.C., O'Roak, B.J., Lozovaya, N., Bruneau, N., et al. (2013). GRIN2A mutations cause epilepsy-aphasia spectrum disorders. *Nat Genet.* 45: 1073-1076. doi: 10.1038/ng.2727.

Chen, W., Tankovic, A., Burger, P.B., Kusumoto, H., Traynelis, S.F., and Yuan, H. (2017). Functional Evaluation of a De Novo GRIN2A Mutation Identified in a Patient with Profound Global Developmental Delay and Refractory Epilepsy. *Mol Pharmacol.* 91: 317-330. doi: 10.1124/mol.116.106781.

Dazzo, E., Rehberg, K., Michelucci, R., Passarelli, D., Boniver, C., Vianello Dri, V., et al. (2018). Mutations in MICAL-1cause autosomal-dominant lateral temporal epilepsy. *Ann Neurol.* 83: 483-493. doi: 10.1002/ana.25167.

de Ligt, J., Willemsen, M.H., van Bon, B.W., Kleefstra, T., Yntema, H.G., Kroes, T., et al. (2012). Diagnostic exome sequencing in persons with severe intellectual disability. *N Engl J Med.* 367: 1921-1929. doi: 10.1056/NEJMoa1206524.

DeVries, S.P., and Patel, A.D. (2013). Two patients with a GRIN2A mutation and childhood-onset epilepsy. *Pediatr Neurol.* 49: 482-485. doi: 10.1016/j.pediatrneurol.2013.08.023.

Endele, S., Rosenberger, G., Geider, K., Popp, B., Tamer, C., Stefanova, I., et al. (2010). Mutations in GRIN2A and GRIN2B encoding regulatory subunits of NMDA receptors cause variable neurodevelopmental phenotypes. *Nat Genet.* 42: 1021-1026. doi: 10.1038/ng.677.

Fainberg, N., Harper, A., Tchapyjnikov, D., and Mikati, M.A. (2016). Response to immunotherapy in a patient with Landau-Kleffner syndrome and GRIN2A mutation. *Epileptic Disord.* 18: 97-100. doi: 10.1684/epd.2016.0791.

Gao, K., Tankovic, A., Zhang, Y., Kusumoto, H., Zhang, J., Chen, W., et al. (2017). A de novo loss-of-function GRIN2A mutation associated with childhood focal epilepsy and acquired epileptic aphasia. *PLoS One.* 12: e0170818. doi: 10.1371/journal.pone.0170818.

Hesse, A.N., Bevilacqua, J., Shankar, K., and Reddi, H.V. (2018). Retrospective genotype-phenotype analysis in a 305 patient cohort referred for testing of a targeted epilepsy panel. *Epilepsy Res.* 144: 53-61. doi: 10.1016/j.eplepsyres.2018.05.004.

Lemke, J.R., Lal, D., Reinthaler, E.M., Steiner, I., Nothnagel, M., Alber, M., et al. (2013). Mutations in GRIN2A cause idiopathic focal epilepsy with rolandic spikes. *Nat Genet.* 45: 1067-1072. doi: 10.1038/ng.2728.

Lesca, G., Rudolf, G., Bruneau, N., Lozovaya, N., Labalme, A., Boutry-Kryza, N., et al. (2013). GRIN2A mutations in acquired epileptic aphasia and related childhood focal epilepsies and encephalopathies with speech and language dysfunction. *Nat Genet.* 45: 1061-1066. doi: 10.1038/ng.2726.

Lindy, A.S., Stosser, M.B., Butler, E., Downtain-Pickersgill, C., Shanmugham, A., Retterer, K., et al. (2018). Diagnostic outcomes for genetic testing of 70 genes in 8565 patients with epilepsy and neurodevelopmental disorders. *Epilepsia.* 59: 1062-1071. doi: 10.1111/epi.14074.

Lionel, A.C., Costain, G., Monfared, N., Walker, S., Reuter, M.S., Hosseini, S.M., et al. (2018). Improved diagnostic yield compared with targeted gene sequencing panels suggests a role for whole-genome sequencing as a first-tier genetic test. *Genet Med.* 20: 435-443. doi: 10.1038/gim.2017.119.

Marwick, K.F.M., Skehel, P.A., Hardingham, G.E., and Wyllie, D.J.A. (2019a). The human NMDA receptor GluN2A(N615K) variant influences channel blocker potency. *Pharmacol Res Perspect.* 7: e00495. doi: 10.1002/prp2.495.

Miao, P., Feng, J., Guo, Y., Wang, J., Xu, X., Wang, Y., et al. (2018). Genotype and phenotype analysis using an epilepsy-associated gene panel in Chinese pediatric epilepsy patients. *Clin Genet.* 94: 512-520. doi: 10.1111/cge.13441.

Monies, D., Abouelhoda, M., AlSayed, M., Alhassnan, Z., Alotaibi, M., Kayyali, H., et al. (2017). The landscape of genetic diseases in Saudi Arabia based on the first 1000 diagnostic panels and exomes. *Hum Genet.* 136: 921-939. doi: 10.1007/s00439-017-1821-8.

Ogden, K.K., Chen, W., Swanger, S.A., McDaniel, M.J., Fan, L.Z., Hu, C., et al. (2017). Molecular Mechanism of Disease-Associated Mutations in the Pre-M1 Helix of NMDA Receptors and Potential Rescue Pharmacology. *PLoS Genet.* 13: e1006536. doi: 10.1371/journal.pgen.1006536.

Retterer, K., Juusola, J., Cho, M.T., Vitazka, P., Millan, F., Gibellini, F., et al. (2016). Clinical application of whole-exome sequencing across clinical indications. *Genet Med.* 18: 696-704. doi: 10.1038/gim.2015.148.

Serraz, B., Grand, T., and Paoletti, P. (2016). Altered zinc sensitivity of NMDA receptors harboring clinically-relevant mutations. *Neuropharmacology.* 109: 196-204. doi: 10.1016/j.neuropharm.2016.06.008.

Singh, D., Lau, M., Ayers, T., Singh, Y., Akingbola, O., Barbiero, L., et al. (2016). De Novo Heterogeneous Mutations in SCN2A and GRIN2A Genes and Seizures With Ictal Vocalizations. *Clin Pediatr (Phila).* 55: 867-870. doi: 10.1177/0009922815601060.

Snoeijen-Schouwenaars, F.M., van Ool, J.S., Verhoeven, J.S., van Mierlo, P., Braakman, H.M.H., Smeets, E.E., et al. (2019). Diagnostic exome sequencing in 100 consecutive patients with both epilepsy and intellectual disability. *Epilepsia.* 60: 155-164. doi: 10.1111/epi.14618.

Strehlow, V., Heyne, H.O., Vlaskamp, D.R.M., Marwick, K.F.M., Rudolf, G., de Bellescize, J., et al. (2019). GRIN2A-related disorders: genotype and functional consequence predict phenotype. *Brain.* 142: 80-92. doi: 10.1093/brain/awy304.

Swanger, S.A., Chen, W., Wells, G., Burger, P.B., Tankovic, A., Bhattacharya, S., et al. (2016). Mechanistic Insight into NMDA Receptor Dysregulation by Rare Variants in the GluN2A and GluN2B Agonist Binding Domains. *Am J Hum Genet.* 99: 1261-1280. doi: 10.1016/j.ajhg.2016.10.002.

Venkateswaran, S., Myers, K.A., Smith, A.C., Beaulieu, C.L., Schwartzentruber, J.A., Consortium, F.C., et al. (2014). Whole-exome sequencing in an individual with severe global developmental delay and intractable epilepsy identifies a novel, de novo GRIN2A mutation. *Epilepsia.* 55: e75-79. doi: 10.1111/epi.12663.

von Stulpnagel, C., Ensslen, M., Moller, R.S., Pal, D.K., Masnada, S., Veggiotti, P., et al. (2017). Epilepsy in patients with GRIN2A alterations: Genetics, neurodevelopment, epileptic phenotype and response to anticonvulsive drugs. *Eur J Paediatr Neurol.* 21: 530-541. doi: 10.1016/j.ejpn.2017.01.001.

Xu, X.X., Liu, X.R., Fan, C.Y., Lai, J.X., Shi, Y.W., Yang, W., et al. (2018). Functional Investigation of a GRIN2A Variant Associated with Rolandic Epilepsy. *Neurosci Bull.* 34: 237-246. doi: 10.1007/s12264-017-0182-6.

Yang, X., Qian, P., Xu, X., Liu, X., Wu, X., Zhang, Y., et al. (2018). GRIN2A mutations in epilepsy-aphasia spectrum disorders. *Brain Dev.* 40: 205-210. doi: 10.1016/j.braindev.2017.09.007.

Yuan, H., Hansen, K.B., Zhang, J., Pierson, T.M., Markello, T.C., Fajardo, K.V., et al. (2014). Functional analysis of a de novo GRIN2A missense mutation associated with early-onset epileptic encephalopathy. *Nat Commun.* 5: 3251. doi: 10.1038/ncomms4251.
