# Supplementary material for: GRIN2A Variants Associated With Idiopathic Generalized Epilepsies
Source: Front Mol Neurosci. 2021 Oct 14;14:720984. doi: 10.3389/fnmol.2021.720984 (PMC8551482; doi:10.3389/fnmol.2021.720984)
Supplement: Supplementary file 4 [file Table_4.docx]

**Supplementary data 4. Phenotypes of epilepsy-related *GRIN2A* missense mutations with loss of function**

|  | **Mutations** | **Locations** | **Functional alteration**  **(glutamate potency)** | **Epileptic syndrome** | **Phenotypic severity** | **Reference** |
| --- | --- | --- | --- | --- | --- | --- |
|  | c.1553G>A/p.R518H | S1 | LOF (complete) | LKS/CSWSS/ABPE | Intermediate | Swanger *et al.*, 2016 |
|  | c.1592C>T/p.T531M | S1 | LOF (complete) | CSWSS | Intermediate | Swanger *et al.*, 2016 |
|  | c.2191G>A/p.D731N | S2 | LOF (complete) | ABPE | Intermediate | Gao *et al.*, 2017; Swanger *et al.*, 2016 |
|  | c.2054T>G/p. V685G | S2 | LOF (79-fold) | EE | Severe | Swanger *et al.*, 2016 |
|  | c.1447G>A/p.G483R | S1 | LOF (15.9-fold/26.2-fold) | CSWSS/ABPE | Intermediate | Swanger *et al.*, 2016; Addis *et al.*, 2017 |
|  | c.2146G>A/p.A716T | S2 | LOF (5.9-fold) | ABPE/BECTS | Intermediate | Swanger *et al.*, 2016 |
|  | c.1306T>C/p.C436R | S1 | LOF (trafficking defect) | ABPE | Intermediate | Swanger *et al.*, 2016 |
|  | c.692G>A/p.C231Y | ATD | LOF (4.5-fold) | LKS | Intermediate | Addis *et al.*, 2017 |
|  | c.1642G>A/p.A548T | S1-M1 | LOF (4.1-fold) | LKS | Intermediate | Ogden *et al.*, 2017 |
|  | c.236C>G/p.P79R | ATD | LOF (3.5-fold) | CSWSS | Intermediate | Addis *et al.*, 2017 |
|  | c.2081T>C/p.I694T | S2 | LOF (2.9-fold) | LKS | Intermediate | Swanger *et al.*, 2016 |
|  | c.2113A>G/p.M705V | S2 | LOF (1.7-fold/2.1-fold) | ABPE | Intermediate | Swanger *et al.*, 2016; Addis *et al.*, 2017 |
|  | c.2179G>A/p.A727T | S2 | LOF (1.5-fold) | BECTS | Mild | Swanger *et al.*, 2016 |
|  | c.2200G>C/p.V734L | S2 | LOF (1.5-fold) | BECTS | Mild | Swanger *et al.*, 2016 |
|  | c.2314A>G/p.K772E | S2 | LOF (1.4-fold) | ABPE | Intermediate | Swanger *et al.*, 2016 |
|  | c.1108C>T/p.R370W | ATD | LOF (Zn^2+^ inhibition increased) | BECTS | Mild | Serraz *et al.*., 2016 |

**Abbreviations:** ATD: amino terminal domain; LBD-S1: ligand-binding domain S1; LBD-S2: ligand-binding domain S2; M1, M3, and M4: transmembrane domains M1, M3, and M4 regions, M2: re-entrant pore loop; CTD: carboxyl-terminal domain; NA: not available; BECTS: benign epilepsy with centro-temporal spikes; ABPE: atypical benign partial epilepsy; CSWSS: continuous spike and waves during sleep; LKS: Landau-Kleffner syndrome; EE: epileptic encephalopathy; EOEE: early onset epileptic encephalopathy; LOF: loss of function.
